# Accurate PROTAC-targeted degradation prediction with DegradeMaster

**DOI:** 10.1093/bioinformatics/btaf191

**Published:** 2025-07-15

**Authors:** Jie Liu, Michael J Roy, Luke Isbel, Fuyi Li

**Affiliations:** South Australian Immunogenomics Cancer Institute (SAiGENCI), The University of Adelaide, Adelaide, South Australia 5005, Australia; South Australian Immunogenomics Cancer Institute (SAiGENCI), The University of Adelaide, Adelaide, South Australia 5005, Australia; South Australian Immunogenomics Cancer Institute (SAiGENCI), The University of Adelaide, Adelaide, South Australia 5005, Australia; Adelaide Centre for Epigenetics, The University of Adelaide, Adelaide, South Australia 5005, Australia; South Australian Immunogenomics Cancer Institute (SAiGENCI), The University of Adelaide, Adelaide, South Australia 5005, Australia

## Abstract

**Motivation:**

Proteolysis-targeting chimeras (PROTACs) are heterobifunctional molecules that can degrade “undruggable” protein of interest by recruiting E3 ligases and hijacking the ubiquitin-proteasome system. Some efforts have been made to develop deep learning-based approaches to predict the degradation ability of a given PROTAC. However, existing deep learning methods either simplify proteins and PROTACs as 2D graphs by disregarding crucial 3D spatial information or exclusively rely on limited labels for supervised learning without considering the abundant information from unlabeled data. Nevertheless, considering the potential to accelerate drug discovery, it is critical to develop more accurate computational methods for PROTAC-targeted protein degradation prediction.

**Results:**

This study proposes DegradeMaster, a semisupervised E(3)-equivariant graph neural network-based predictor for targeted degradation prediction of PROTACs. DegradeMaster leverages an E(3)-equivariant graph encoder to incorporate 3D geometric constraints into the molecular representations and utilizes a memory-based pseudolabeling strategy to enrich annotated data during training. A mutual attention pooling module is also designed for interpretable graph representation. Experiments on both supervised and semisupervised PROTAC datasets demonstrate that DegradeMaster outperforms state-of-the-art baselines, with substantial improvement of AUROC by 10.5%. Case studies show DegradeMaster achieves 88.33% and 77.78% accuracy in predicting the degradability of VZ185 candidates on BRD9 and ACBI3 on KRAS mutants. Visualization of attention weights on 3D molecule graph demonstrates that DegradeMaster recognizes linking and binding regions of warhead and E3 ligands and emphasizes the importance of structural information in these areas for degradation prediction. Together, this shows the potential for cutting-edge tools to highlight functional PROTAC components, thereby accelerating novel compound generation.

**Availability and implementation:**

The source code and datasets are available at https://github.com/ABILiLab/DegradeMaster and https://zenodo.org/records/14715718.

## 1 Introduction

Proteolysis-targeting chimeras (PROTACs) are heterobifunctional molecules comprising a target protein-of-interest (POI) binding moiety (warhead), a linker, and an E3 ubiquitin ligase binding moiety (Sakamoto *et al.* 2001, Toure and Crews 2016). By forming a ternary POI–PROTAC–E3 ligase complex (Neklesa *et al.* 2017, Pettersson and Crews 2019), PROTACs exploit the ubiquitin-proteasome system to induce polyubiquitination and subsequent proteasomal degradation of the target protein (Zhao *et al.* 2022). As an emerging therapeutic strategy, PROTACs offer advantages over traditional small-molecule inhibitors that are attractive to target previously “undruggable” proteins (Békés *et al.* 2022), including addressing noncatalytic functions of target proteins, potential for enhanced selectivity and specificity, and preventing the accumulation of drug targets (Lopez-Girona *et al.* 2012, Liu *et al.* 2022).

Currently, PROTAC development is highly dependent on iterative strategies for design, synthesis, biological evaluation, and optimization (Hu *et al.* 2019, Zoppi *et al.* 2018), which can be time-intensive and costly. Thanks to the increasing availability of detailed and high-quality PROTAC data (Gadd *et al.* 2017, Farnaby *et al.* 2019, Hartung *et al.* 2023) and extensive PROTAC databases like PROTAC-DB (Ge *et al.* 2025), machine learning has emerged as a viable tool for predicting PROTAC degradation. Traditional machine learning models, such as support vector machine (SVM) (Cortes 1995) and random forest (RF) (Ho 1995), leverage physicochemical descriptors of PROTAC molecules and target proteins for predictive modeling (Zhang *et al.* 2022, Xie and Xie 2023). Recent research has advanced the use of graphs to model the complex structural intricacies of the PROTAC system. Deep learning approaches, including graph neural networks (GNNs) (Kipf and Welling 2016), long short-term memory networks (Hochreiter 1997), and transformers (Vaswani 2017), have been employed to encode both structural and attribute-based information of PROTAC molecules and target proteins, facilitating downstream tasks such as degradation prediction (Li *et al.* 2022, Kao *et al.* 2023, Chen *et al.* 2024).

Despite their notable performance, deep learning models face two major limitations: first, existing deep learning approaches (Li *et al.* 2022, Chen *et al.* 2024) represent proteins and PROTACs as 2D graphs for simplicity, disregarding their 3D atomic coordinates. However, the 3D spatial arrangement of atoms is crucial for determining binding interactions and molecular geometry, which directly influence the efficacy of PROTAC-mediated target protein degradation. The omission of 3D spatial information leads to the loss of structural compatibility, thereby diminishing model accuracy. Second, existing predictive methods exclusively rely on supervised learning, yet the datasets from PROTAC-DB are predominantly unlabeled, with over 80% of the data lacking annotation of degradation activity (i.e. lacking values for $DC_{50}$ and *D*_max_, respectively, the half-maximal degradation concentration and percentage maximum degradation achieved). This scarcity of labeled data increases the risk of overfitting, particularly when employing complex model architectures. Consequently, developing a semisupervised learning approach that incorporates the abundant unlabeled data into the training process offers a promising solution to mitigate the issue of limited annotations and improve prediction accuracy.

To address these challenges, we propose a semisupervised E(3)-equivariant graph neural network (DegradeMaster) for PROTAC degradation prediction. DegradeMaster integrates five key components: (i) a 3D molecule graph construction module to capture spatial and structural information from the PROTAC system, (ii) an E(3)-equivariant encoder to incorporate geometric constraints such as distances and angles into the molecular representations, (iii) a feature selection module to extract informative chemical descriptors as attribute features, (iv) a mutual attention pooling module to compute comprehensive graph representations, and (v) a memory-based pseudolabeling module to expand the size of annotated data during training. Benchmarking experiments on two PROTAC-DB dataset variants demonstrate that DegradeMaster outperforms PROTAC-STAN (Chen *et al.* 2024) and other state-of-the-art models (Li *et al.* 2022) for PROTAC degradation prediction.

## 2 Materials and methods

### 2.1 Data collection and preprocessing

To evaluate the performance of our model and baseline methods, we utilized data from the PROTAC-DB 3.0 database (Ge *et al.* 2025). The latest release of PROTAC-DB 3.0 comprises 9380 PROTAC entries, including 569 warheads, 107 E3 ligands, and 5753 linkers. Each entry includes detailed information such as the PROTAC’s SMILES representation, and UniProt ID of the POI and the E3 ligase. Additionally, the database provides data on PROTAC degradation activity, including $DC_{50}$ and *D*_max_. These metrics serve as standard measures of a PROTAC’s efficiency in degrading target proteins (Pettersson and Crews 2019, Liu *et al.* 2022), where a lower $DC_{50}$ and a higher *D*_max_ indicate relatively high degradation potential.

We first removed entries that lack critical information, e.g. the UniProt ID of the POI or E3 ligase. For degradation labels, following Li *et al.* (2022), we utilized both explicit $DC_{50}$/$D_{\max}$ values and implicit values inferred from experimental descriptions to predict PROTAC degradation activity. A PROTAC is considered to have low degradation activity if $DC_{50}$ is greater than or equal to 100 nM and *D*_max_ is below 80%, otherwise it is labeled with high degradation activity. Crystal structures of POIs and E3 ligases are sourced from the Protein Data Bank (Berman *et al.* 2000), while proteins without available crystal structures are supplemented with predicted structures generated by AlphaFold 2 (Jumper *et al.* 2021). We applied Smina (Koes *et al.* 2013) to dock the warhead and E3 ligand to POI and E3 ligase, respectively, defining the residues within 5 Å of the ligand as the pocket. Using these criteria, we constructed a supervised PROTAC dataset consisting of 620 high-activity entries and 1011 low-activity entries. Additionally, we curated a semisupervised PROTAC dataset containing 8603 entries in total, incorporating the same labeled subset as the supervised dataset. Dataset information is elaborated in Table 1.

**Table 1.** Statistics of the two PROTAC datasets extracted from PROTAC-DB 3.0 (Ge *et al.* 2025).^a^

Datasets	Entries	Warheads	E3-ligands	Linkers	High	Low
PROTAC-1K	1503	360	80	1500	577	925
PROTAC-8K	8636	569	107	5753	577	925

a High and low indicate high and low degradation activities of PROTACs, respectively.

### 2.2 The framework of DegradeMaster

**Figure 1.**
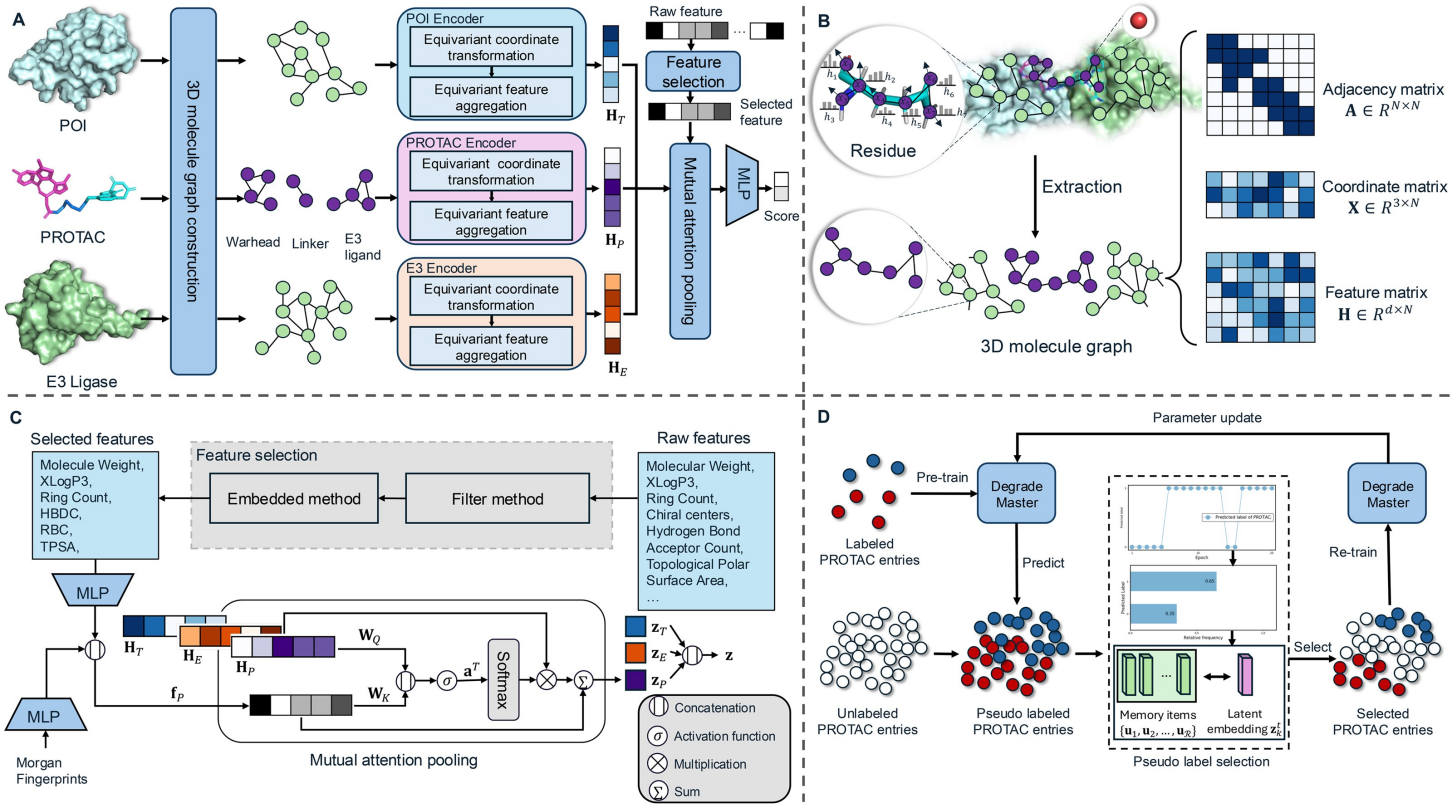
(A) Overall framework of DegradeMaster. (B) 3D molecule graph construction procedure. (C) Feature selection and mutual attention pooling. Abbreviations: HBDC, hydrogen bond donor count; RBC, rotatable bond count; TPSA, topological polar surface area. (D) Memory-enhanced pseudolabeling.

An overview of the architecture of DegradeMaster and its key components are illustrated in Fig. 1. As depicted in Fig. 1A, DegradeMaster employs a multistep framework designed to predict the degradation efficacy of PROTAC molecules by effectively capturing spatial, structural, and physicochemical information. It consists of five major components: 3D molecule graph construction, E(3) equivariant encoder, feature selection, mutual attention pooling, and label enrichment.

To refine the input data, a feature selection module identifies the most informative molecular properties, filtering out redundant attributes or those that do not influence the model greatly. A two-step process, combining statistical metrics such as variance and chi-square scores with an embedded method using Gradient Boosting Decision Trees (Friedman, 2001), is applied. As a result, six out of the 11 chemical descriptors are selected. These six chemical properties, along with the Morgan fingerprints, are then fed into two independent one-layer multilayer perceptrons (MLPs). The outputs from both networks are concatenated to form the attribute vector $f_{P}$. A detailed explanation of feature selection is provided in Section 1 of the Supplementary Material.

The other four components of the methodology will be elaborated in the following section.

#### 2.2.1 3D molecule graph construction

In this section, we represent the spatial and structural information of molecules using graphs. As illustrated in the top-left of Fig. 1B, a PROTAC molecule is modeled with its atoms as nodes and chemical bonds as edges, forming the node set $V_{P}$ and the edge set $E_{P}$, respectively. Proteins, specifically the POI and E3 ligase, are divided into residues (depicted as dots outside the circle in Fig. 1B), with each residue comprising multiple atoms (shown as dots inside the circle). The node sets for the POI and E3 ligase graphs are denoted as $V_{T}$ and $V_{E}$, respectively, where each node $v\in V_{T}$ or $V_{E}$ corresponds to an atom. The edge sets are defined as $E_{\zeta }=\{E_{\zeta ,\text{intra}},E_{\zeta ,\text{inter}}\}$ for ζ∈{T,E}, where $E_{\cdot ,\text{intra}}$ and $E_{\cdot ,\text{inter}}$ represent intraresidue and interresidue connections, respectively. For each molecule graph $G_{\zeta }$ comprising $N_{\zeta }$ atoms, the adjacency matrix is represented as $A_{\zeta }\in R^{N_{\zeta }\times N_{\zeta }}$, the feature matrix as $H_{\zeta }\in R^{N_{\zeta }\times d_{\zeta }}$ (where the *p*th row of $H_{\zeta }$ encodes the feature vector of atom *p*), and the 3D atom coordinates as $X_{\zeta }\in R^{N_{\zeta }\times 3}$. In summary, the constructed 3D molecule graph $G_{\zeta }$ is defined as $G_{\zeta }=\{A_{\zeta },X_{\zeta },H_{\zeta }\}$ for ζ∈{P,T,E}.

#### 2.2.2 E(3) equivariance

In this section, we introduce the concept of E(3) equivariance, which serves as a crucial inductive bias for predicting PROTAC degradation efficacy. The Euclidean group E(3) consists of transformations in 3D space that preserve the Euclidean distance between any two points (Miller 1973, Cornwell 1997). Let X represent the coordinate matrix of the input 3D molecule graph G, and $T_{G}:X$ denote a linear transformation applied to X. A graph encoder $f_{\phi }:X\to Y$ is defined as E(3) equivariant to G if there exists a corresponding transformation $S_{G}:Y\to Y$ in the output space such that the following condition holds (Satorras *et al.* 2021):

(1) $$S_{G}(f_{\phi }(X))=f_{\phi }(T_{G}(X)).$$

Specifically, let the linear transformation be $T_{G}(X)=\mathrm{MX}+B$, the equivalent transformation is $S_{G}(Y)=\mathrm{MY}+B$, where $M\in R^{N\times N}$ denotes the rotation and reflection transition, while $B\in R^{N\times N}$ denotes the translation transition. This ensures that the encoder output transforms consistently with the input under any E(3) transformation, preserving spatial and structural relationships critical for accurate degradation efficacy prediction.

#### 2.2.3 E(3) equivariant encoder

In this section, we elaborate on the design of our E(3) equivariant graph encoder. As shown in Fig. 1A, the equivariant graph encoder consists of two key steps: equivariant coordinate transformation and equivariant feature aggregation. We adopt the equivariant graph neural network (Satorras *et al.* 2021) as the base model in our coordinate update step. In the following part, we will introduce the two steps separately.

**Equivariant coordinate transformation.** Following the notion defined in Section 2.2.1, given a 3D molecule graph $G_{\zeta }=\{A_{\zeta },X_{\zeta },H_{\zeta }\}$ for ζ∈{P,T,E}, each node $v_{i}\in V_{\zeta }$ is associated with a coordinate vector $x_{i}\in R^{3}$ and a feature vector $h_{i}\in R^{d_{\zeta }}$. Let $x_{i}^{\left(l-1\right)}$ and $h_{i}^{\left(l-1\right)}$ denote the coordinate embedding and feature embedding of node $v_{i}$ after (l−1)-layer encoding, $e_{ij}\in E_{\zeta }$ is the edge connecting to $v_{i}$, and the coordinate vector $x_{i}^{\left(l\right)}$ is calculated as follows:

(2) $$m_{ij}=\phi_{e}\left(a_{ij},h_{i}^{\left(l-1\right)},h_{j}^{\left(l-1\right)},\left||x_{i}^{\left(l-1\right)}-x_{j}^{\left(l-1\right)}\right|{|}^{2}\right),$$

 

(3) $$\Delta x_{i}^{\left(l\right)}=\sum_{j=1}^{n_{i}}\frac{x_{i}^{\left(l-1\right)}-x_{j}^{\left(l-1\right)}}{\left||x_{i}^{\left(l-1\right)}-x_{j}^{\left(l-1\right)}\right||}\phi_{x}(m_{ij}),$$

 

(4) $$x_{i}^{\left(l\right)}=x_{i}^{\left(l-1\right)}+\Delta x_{i}^{\left(l\right)},$$

where $a_{ij}$ is the edge attribute, $\phi_{e}(\cdot )$ and $\phi_{x}(\cdot )$ are MLPs that compute messages for edge and coordinate representations, respectively. $n_{i}$ denotes the number of neighbors of node $v_{i}$.

**Equivariant feature aggregation.** The vector $m_{ij}$ computed in Equation (2) indicates the message passed through edge $e_{ij}$. Thus, the feature aggregation on node embedding $h_{i}^{\left(l\right)}$ can be formed as follows:

(5) $$m_{i}=\sum_{j=1}^{n_{i}}m_{ij},$$

 

(6) $$h_{i}^{\left(l\right)}=\phi_{h}\left(h_{i}^{\left(l-1\right)}\mathrm{||}m_{i}\right),$$

where $\phi_{h}(\cdot )$ is the MLP for node feature aggregation. By varying the input graphs among $G_{P}$, $G_{T}$, and $G_{E}$, we obtain node embeddings $H_{P}$, $H_{T}$, and $H_{E}$ for PROTAC graph, POI graph, and E3 ligase graph in E(3)-equivariant manner, respectively.

#### 2.2.4 Mutual attention pooling

In Section 2.2.3, we incorporate the spatial and structural information of molecules and encode node embeddings in an E(3)-equivariant manner. Using the encoded node embeddings for the PROTAC molecule, $H_{P}\in R^{N_{P}\times d^{\prime }}$, we apply a pooling operation to obtain the representation of the entire PROTAC graph. Unlike the mean pooling strategy employed in prior works (Li *et al.* 2022), we introduce a mutual attention-based pooling strategy. This approach calculates contribution weights for each node’s relevance to degradation and applies weighted pooling accordingly. The PROTAC graph embedding $z_{P}\in R^{d^{\prime }}$ is computed as follows:

(7) $$q_{i}=W_{Q}h_{\left(i,P\right)},$$

 

(8) $$k_{P}=W_{K}f_{P},$$

 

(9) $$w_{\left(i,P\right)}=\text{Softmax}\left(a^{T}\cdot \sigma (q_{i}∥k_{P})\right),$$

 

(10) $$z_{P}=\sum_{i=1}^{N_{P}}w_{\left(i,P\right)}h_{\left(i,P\right)}+f_{P},$$

where $W_{Q}\in R^{d^{\prime }\times d_{a}}$ and $W_{K}\in R^{d_{f}\times d_{a}}$ are projection matrices, with $d^{\prime }$, $d_{f}$, and $d_{a}$ representing the feature dimensions of node embeddings, selected molecular attributes, and attention, respectively. $h_{\left(i,P\right)}\in R^{d^{\prime }}$ is the embedding of node *i*, and $f_{P}\in R^{d_{f}}$ is the vector of selected attributes for the PROTAC graph. The trainable vector $a\in R^{2d_{a}}$ serves as the attention vector, and σ(·) is the activation function, where we utilize tanh(·). Using the same procedure, the graph embeddings $z_{T}$ and $z_{E}$ for POI and E3 ligase graphs are computed. The final representation z for the POI–PROTAC–E3 ligase ternary complex is obtained by concatenating the three graph embeddings.

#### 2.2.5 Label enrichment

As discussed in Section 2.1, only 1502 samples out of the total 8603 PROTAC entries in the collected dataset are labeled. Thus, employing a semisupervised learning approach that integrates a large volume of unlabeled data into the training process is advantageous for addressing the lack of annotated samples and enhancing prediction accuracy (Liu *et al.* 2022). Inspired by recent advancements in pseudolabeling based on training dynamics (Pei *et al.* 2024), we propose a memory-enhanced pseudolabeling strategy to generate and select pseudolabels using a combined selection score. Specifically, we adopt the disagreement score design from MoDis (Pei *et al.* 2024) and propose a novel memory-based score.

Following the process proposed in Pei *et al.* (2024), we compute the disagreement score by first pretraining the DegradeMaster model on labeled samples to predict the labels of unlabeled data. As illustrated in Fig. 1D, for each unlabeled entry *k*, the predicted labels across selected training epochs form the pseudolabel set M. The logits $z_{k}$ are used to compute the prediction distribution $P_{k}(c)$, reflecting the relative frequency of class *c*. The disagreement score $s_{\mathrm{dis}}(k)$, calculated as the cross-entropy of $P_{k}(c)$, measures the consistency of predictions for *k*. A higher $s_{\mathrm{dis}}(k)$ indicates a stronger candidate for pseudolabeling.

To refine pseudolabel selection further, we design a memory-based score using latent feature similarity. We record the latent embedding $z_{i}^{t}$ for each labeled entry $i\in U_{l}$ at epoch t∈T, and calculate the memory prototype $u_{r}\in R^{d^{\prime }}(r\in 1,\ldots R)$ for the labeled data. Here, R is a hyperparameter determining the number of prototypes, and $u_{r}$ is computed as follows:

(11) $$w_{\left(i,r\right)}=\frac{\;\text{exp}(d(z_{i}^{t},u_{r}))}{\sum_{j=1}^{\left|S_{l}\right|}\;\text{exp}\;(d(h_{i}^{t},u_{j}))},$$

 

(12) $$d(z_{i}^{t},u_{r})=\frac{u_{r}^{T}z_{i}^{t}}{\left\|u_{r}\right\|\left\|z_{i}^{t}\right\|},$$

 

(13) $$u_{r}\leftarrow u_{r}+\sum_{i=1}^{\left|S_{l}\right|}w_{\left(i,r\right)}z_{i}^{t}.$$

The latent embedding of the unlabeled entry $k\in S_{u}$ at epoch t∈T is denoted as $z_{k}^{t}$. The memory-based score $s_{\mathrm{mem}}(\cdot )$ is calculated as the cosine similarity between $z_{k}^{t}$ and the memory prototypes $u_{r}$:

(14) $$s_{\mathrm{mem}}(k)={\text{Max}}_{r=1,\ldots R}\left(\frac{u_{r}^{T}z_{k}^{t}}{\left\|u_{r}\right\|\left\|z_{k}^{t}\right\|}\right).$$

The $s_{\mathrm{mem}}(\cdot )$ score measures the similarity between the latent embedding of unlabeled data and labeled data. Higher $s_{\mathrm{mem}}$ values indicate more promising pseudolabeling candidates. By combining $s_{\mathrm{dis}}(\cdot )$ and $s_{\mathrm{mem}}(\cdot )$, we acquire the combined selection score *s*:

(15) $$s=\frac{1}{2}(s_{\mathrm{dis}}+s_{\mathrm{mem}}).$$

After T training epochs, we rank the unlabeled data based on *s* score and select the top *K* samples with the highest scores. The corresponding predicted labels are chosen from M as pseudolabels, which are then combined with real labels to retrain DegradeMaster. Note that we only use the training set for label enrichment and directly apply the retrained model for prediction on the test set.

## 3 Results and discussion

We use information from the PROTAC-DB 3.0 (Ge *et al.* 2025) as described in Section 2.1, and construct a supervised PROTAC dataset named PROTAC-1K and a semisupervised PROTAC dataset named PROTAC-8K. We randomly split both PROTAC-1K and PROTAC-8k datasets into training/testing sets with an 80/20 ratio. To prevent data leakage, any samples in the test set sharing the same target protein and PROTAC SMILES as those in the training set are excluded. Note that PROTAC-1K and PROTAC-8K share identical labeled data in both their training and testing sets. We trained SVM and RF in a supervised setting using PROTAC-1K and trained three GNN-based models in both supervised and semisupervised settings. Model configuration is shown in Supplementary Table S4. The performance of DegradeMaster was evaluated against four baselines: SVM (Cortes 1995), RF (Ho 1995), DeepPROTACs (Li *et al.* 2022), and PROTAC-STAN (Chen *et al.* 2024) under the same random split setting, using the metrics outlined in Section 2 in the Supplementary Material. For SVM and RF, we utilize auto cross-covariance features for the POI and E3 ligase, while representing the PROTAC molecule using either molecular access system (MACCS) keys (Durant *et al.* 2002) or Morgan fingerprints (Morgan 1965), following the setting outlined in DeepPROTAC (Li *et al.* 2022). For a fair comparison, we apply the same random seed for all models.

### 3.1 Model performance

**Figure 2.**
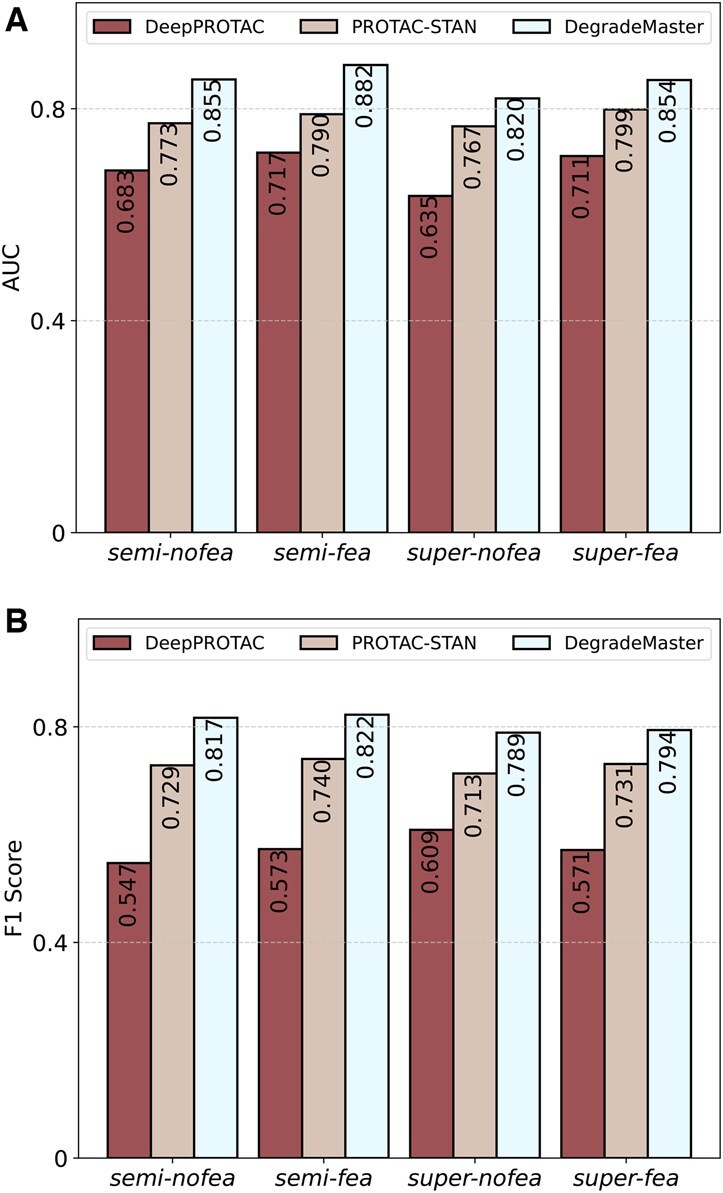
Performance comparison between DegradeMaster and two deep learning baselines (DeepPROTAC and PROTAC-STAN) on the testing dataset: (A) AUC comparison of models under four different settings. (B) F1 comparison of models under four settings. Abbreviations: semi-nofea, semisupervised with no features; semi-fea, semisupervised with features; super-nofea, supervised with no features; super-fea, supervised with features.

**Figure 3.**
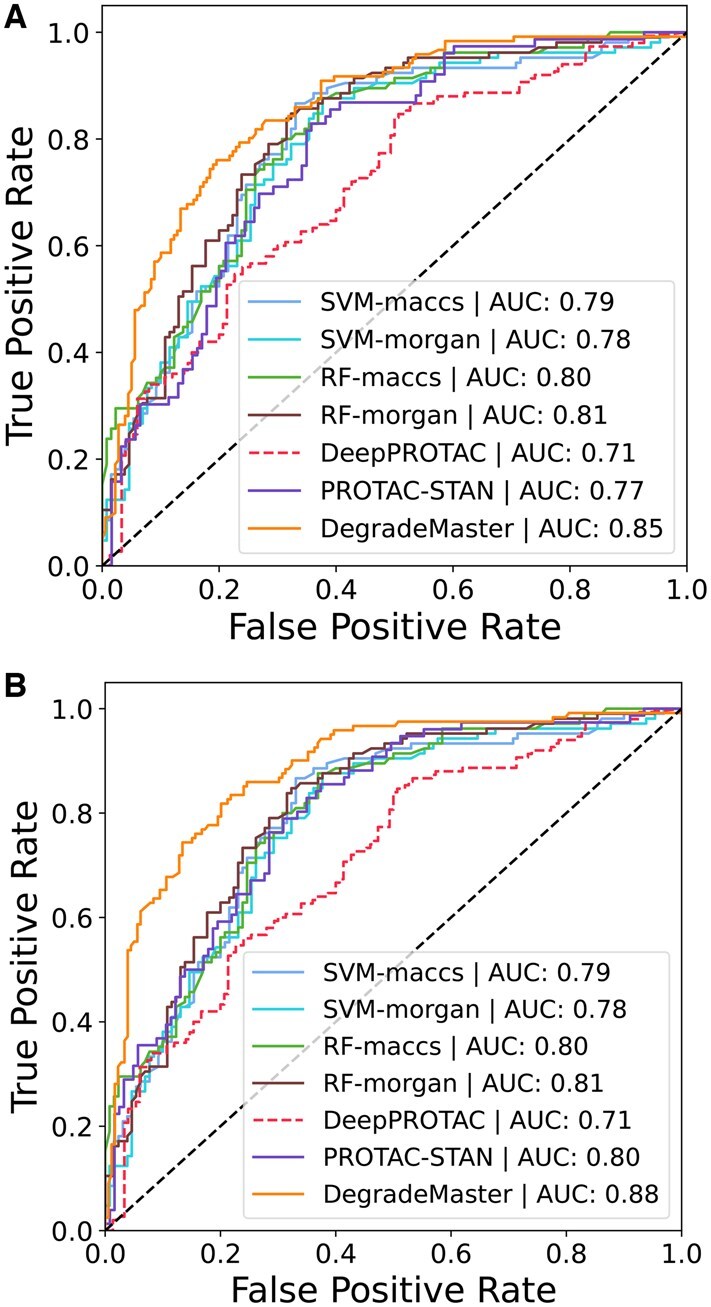
ROC curves of DegradeMaster and compared baselines. (A) ROC curves on PROTAC-1K. (B) ROC curves on PROTAC-8K.

The comparison results are presented in Table 2. Both SVM and RF methods outperform DeepPROTACs across all three metrics. Specifically, SVM achieves a 15.76% improvement in accuracy, while RF shows a 13.64% higher AUROC score compared to DeepPROTAC. These results underscore the efficacy of machine learning approaches, even in their simplicity, particularly when applied to limited training data. Moreover, the fingerprint-based features prove effective, with Morgan fingerprints demonstrating a slight advantage over MACCS fingerprints. The results with and without the raw features are depicted in Fig. 2. The ROC curves of the compared models are shown in Fig. 3. 

**Table 2.** The evaluation results of DegradeMaster and the baseline methods on the test set.^a^

Method	FGP	Setting	Acc (%)	AUROC	F1 score
SVM	MACCS	Super	72.34	0.7815	0.7232
	Morgan	Super	74.47	0.7901	0.7446
RF	MACCS	Super	67.65	0.7981	0.6498
	Morgan	Super	63.40	0.8077	0.5818
DeepPROTACs	–	Super	58.21	0.7107	0.5714
	–	Semi	59.02	0.7171	0.5731
PROTAC-STAN	–	Super	79.34	0.7986	0.7310
	–	Semi	78.25	0.7896	0.7403
DegradeMaster	–	Super	81.41	0.8541	0.8139
	–	Semi	**83.66**	**0.8825**	**0.8224**

a The SVM and RF methods are implemented with two fingerprints, i.e. MACCS and Morgan. In all metrics, DegradeMaster (bold) outperformed alternative models, while the best-performing baselines are underlined. Abbreviations: Acc, accuracy; FGP, Fingerprints, Super, Supervised; Semi, semisupervised.

Both variants of DegradeMaster consistently surpass the existing methods, demonstrating superior performance across accuracy, AUROC, and F1 score. This success is attributed to the E(3)-equivariant design of the graph encoder, which effectively integrates the spatial and structural properties of molecules in 3D space into the representations, thereby enhancing downstream prediction tasks. Specifically, DegradeMaster surpasses the previous state-of-the-art model, PROTAC-STAN, by 2.6% in accuracy, 6.9% in AUROC, and 11.34% in F1 score under the supervised setting. Additionally, it achieves a 6.9% improvement in accuracy, 11.76% in AUROC, and 8.21% in F1 score under the semisupervised setting. These results underscore DegradeMaster’s advanced predictive capabilities and its effectiveness in PROTAC degradation prediction. Furthermore, comparing the performance of the supervised and semisupervised variants of the three deep learning models reveals contrasting trends. Existing models like DeepPROTACs exhibit only marginal performance gains when additional unlabeled PROTAC entries are incorporated, while PROTAC-STAN experiences slight performance degradation. These results demonstrate that incorporating unlabeled data without carefully designed mechanisms for label enhancement does not necessarily improve model generalization or performance. In contrast, DegradeMaster demonstrates a notable improvement of 3.3% in AUROC under the semisupervised setting. This underscores the effectiveness of our label enhancement strategy, which identifies high-quality unlabeled data and generates pseudolabels for inclusion in the training set. By addressing the challenge of label scarcity, this approach enhances the scalability and performance of our model.

### 3.2 Ablation study

In this section, we conduct an ablation study to analyze the contributions of the core components of DegradeMaster, as described in Section 2.2. Table 3 summarizes the results. Five variants of DegradeMaster are evaluated, including four degraded versions and the full model. Each variant isolates specific modules to assess their individual and collective impact. The variants are described as follows:

**Table 3.** Ablation study.

Component	Feature selection	Attention pooling	Equivariant encoder	Label enrichment	Acc (%)	AUROC	F1 score
${\text{DM}}_{\mathrm{raw}}$	✗	✗	✗	✗	70.33	0.8104	0.6987
${\text{DM}}_{w/o\;\;\mathrm{sele}}$	✗	✓	✓	✓	78.33	0.8583	0.7720
${\text{DM}}_{w/o\;\;\mathrm{att}}$	✓	✗	✓	✓	76.33	0.8229	0.7630
${\text{DM}}_{w/o\;e(3)}$	✓	✓	✗	✓	72.66	0.8044	0.7201
${\text{DM}}_{w/o\;\;\mathrm{pseu}}$	✓	✓	✓	✗	80.01	0.8575	0.7891
${\text{DM}}_{\mathrm{full}}$	✓	✓	✓	✓	83.66	0.8825	0.8224

Abbreviation: DM, DegradeMaster.

-
${\text{DegradeMaster}}_{w/o\;\;\mathrm{sele}}$ : This variant removes the feature selection module, using all chemicophysical properties from PROTAC-DB 3.0 as raw input features.
-
${\text{DegradeMaster}}_{w/o\;\;\mathrm{att}}$ : This variant excludes the attention mechanism by replacing attention pooling with mean pooling.
-
${\text{DegradeMaster}}_{w/o\;e(3)}$ : This version substitutes the E(3)-equivariant encoder with a standard GCN (Kipf and Welling 2016) encoder, removing the 3D spatial structural representation.
-
${\text{DegradeMaster}}_{w/o\;\;\mathrm{pseu}}$ : This variant disables the label enrichment strategy by setting *K* to 0, thereby omitting pseudolabeling.
-
${\text{DegradeMaster}}_{\mathrm{raw}}$ : This baseline excludes all key components, including feature selection, attention pooling, the E(3)-equivariant encoder, and label enrichment.
-
${\text{DegradeMaster}}_{\mathrm{full}}$ : Full model of DegradeMaster with all the components available.

From Table 3, it is evident that ${\text{DegradeMaster}}_{\mathrm{raw}}$ demonstrates a significant performance decline compared to ${\text{DegradeMaster}}_{\mathrm{full}}$, with 18.95% decrease in accuracy and 17.7% in F1 score. This underscores the importance of the overall design and the integration of the proposed key components. When comparing ${\text{DegradeMaster}}_{w/o\;e(3)}$ with ${\text{DegradeMaster}}_{\mathrm{full}}$, the largest performance reduction of removing a single component is observed, highlighting the critical role of the E(3)-equivariant encoder in degradation prediction tasks. This finding emphasizes the necessity of incorporating 3D spatial and structural information of proteins and molecules into the representation. The removal of the attention pooling module (${\text{DegradeMaster}}_{w/o\;\;\mathrm{att}}$) leads to a 9.6% decrease in accuracy and a 7.24% reduction in AUROC compared to ${\text{DegradeMaster}}_{\mathrm{full}}$. This result demonstrates the effectiveness of the proposed attention mechanism in selectively aggregating node embeddings in proteins and molecules. The elimination of the pseudolabeling component results in the reduction of accuracy by 4.56% and AUROC by 2.91%. This demonstrates the efficacy of the proposed pseudolabeling strategy in leveraging the rich information from unlabeled data.

### 3.3 Attention weight visualization

**Figure 4.**
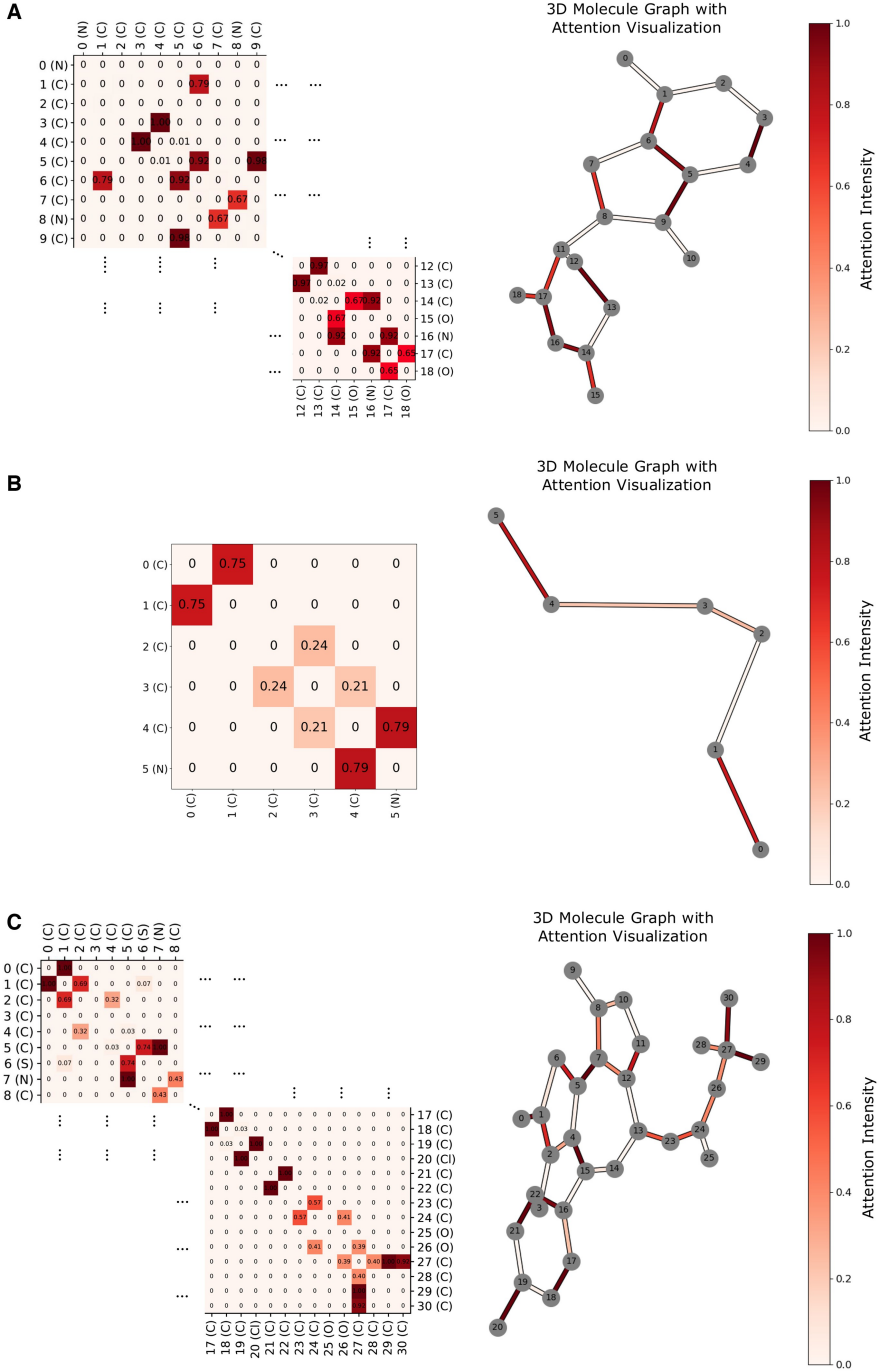
Visualization of attention weights for the PROTAC molecule (PROTAC-DB ID: 194) facilitating degradation of the BRD2 target protein via the CRBN E3 ligase. The left panel displays an attention heatmap among atoms within the molecule, while the right panel presents the 3D molecular graph, where edge color intensity represents attention values. (A) Attention values targeting the E3 ligand. (B) Attention values are associated with the linker. (C) Attention values targeting the warhead.

As described in Section 2.2.4, we implement a mutual attention mechanism during pooling to compute protein-level and molecule-level embeddings. To facilitate interpretability, we visualize the attention weights $w_{\left(i,P\right)}$ by mapping them onto the edges of the PROTAC graph. Specifically, we calculate the average attention weights of node *i* and *j* as $1/2(w_{(i,P)+}w_{\left(j,P\right)})$, and visualize it with the color intensity of the edge $e_{ij}$. This enables a bioinformatic analysis of the significance and distribution of the attention values. Figure 4 illustrates the attention weights among atoms within a PROTAC molecule (Qiu *et al.* 2019) that facilitates the degradation of the BRD2 protein via the CRBN ligase. The 3D molecular graphs are constructed from the atomic coordinates provided in the mol2 file. Based on Fig. 4, we derive the following observations:

*Linking areas show higher attention weights*: Atoms located at the interface regions connecting the warhead- and E3-binding ligand exhibit higher attention weights. For instance, in Fig. 4A, the edge between atom 1 (C) and atom 6 (C) has an attention weight of 0.794, while the edge between atom 6 (C) and atom 5 (C) has an attention weight of 0.917. Similarly, in Fig. 4C, the edges between atom 27 (C) and atoms 28 (C), 29 (C), and 30 (C) have significantly higher weights compared to the central region, such as between atom 13 and atom 14. This highlights the model’s ability to recognize linking regions of target/E3 ligands and emphasizes the importance of structural information in these areas for degradation prediction.

*Ends of the linker have higher attention*: As shown in Fig. 4B, atoms at both ends of the linker display higher attention weights than those in the middle. This aligns with the actual interaction modes between the linker and the target/E3 ligands.

*Binding regions of the POI target and E3 ligands show higher attention*: Atoms at the distal ends of the target and E3 ligands also exhibit elevated attention weights. For example, in Fig. 4C, atoms 20 (Cl) and 19 (C), as well as atoms 18 (C) and 17 (C), show higher attention weights. Similarly, in Fig. 4A, atoms 17 (C) and 16 (N), as well as atoms 16 (N) and 14 (C), demonstrate higher weights. As depicted in Supplementary Fig. S1, these atoms are located near the binding pockets of the POI and E3 ligase (e.g. GLU-187 in the E3 ligase and GLU-735 in the POI). These patterns correspond to the actual binding modes (Pereira *et al.* 2023, Qiang *et al.* 2024), with the warhead binding to the target protein and the E3 ligand interacting with the E3 ligase.

Supplementary Fig. S1 illustrates the binding interactions between the PROTAC molecule and protein pockets. The cyan region represents the CRBN ligase, while the purple region denotes the POI. PROTAC is shown in green. Residues located within 5 Å of the ligase ligand and warhead are selected as the binding pocket of E3 ligase and POI, respectively. The attention weight for each residue is calculated as the mean of the attention weights of the atoms within that residue. Residues with the highest attention weights are labeled in Supplementary Fig. S1A, with their corresponding attention values displayed in Supplementary Fig. S1B and C. Notably, residues PRO-186 and GLU-187 in the ligase pocket, as well as residues GLU-735 and LYS-732 near the binding regions of the ligase ligand and warhead, are identified as high-weight residues, highlighting their importance in the binding interaction.

## 4 Case study

To further demonstrate the predictive capabilities of DegradeMaster, we conduct two case studies. Case study 1 focuses on predicting candidates during the development of the PROTAC VZ185 (Zoppi *et al.* 2018), while Case study 2 evaluates the model’s ability to predict the degradability of PROTAC ACBI3 across different KRAS mutants (Popow *et al.* 2024).

### 4.1 Case study 1

**Figure 5.**
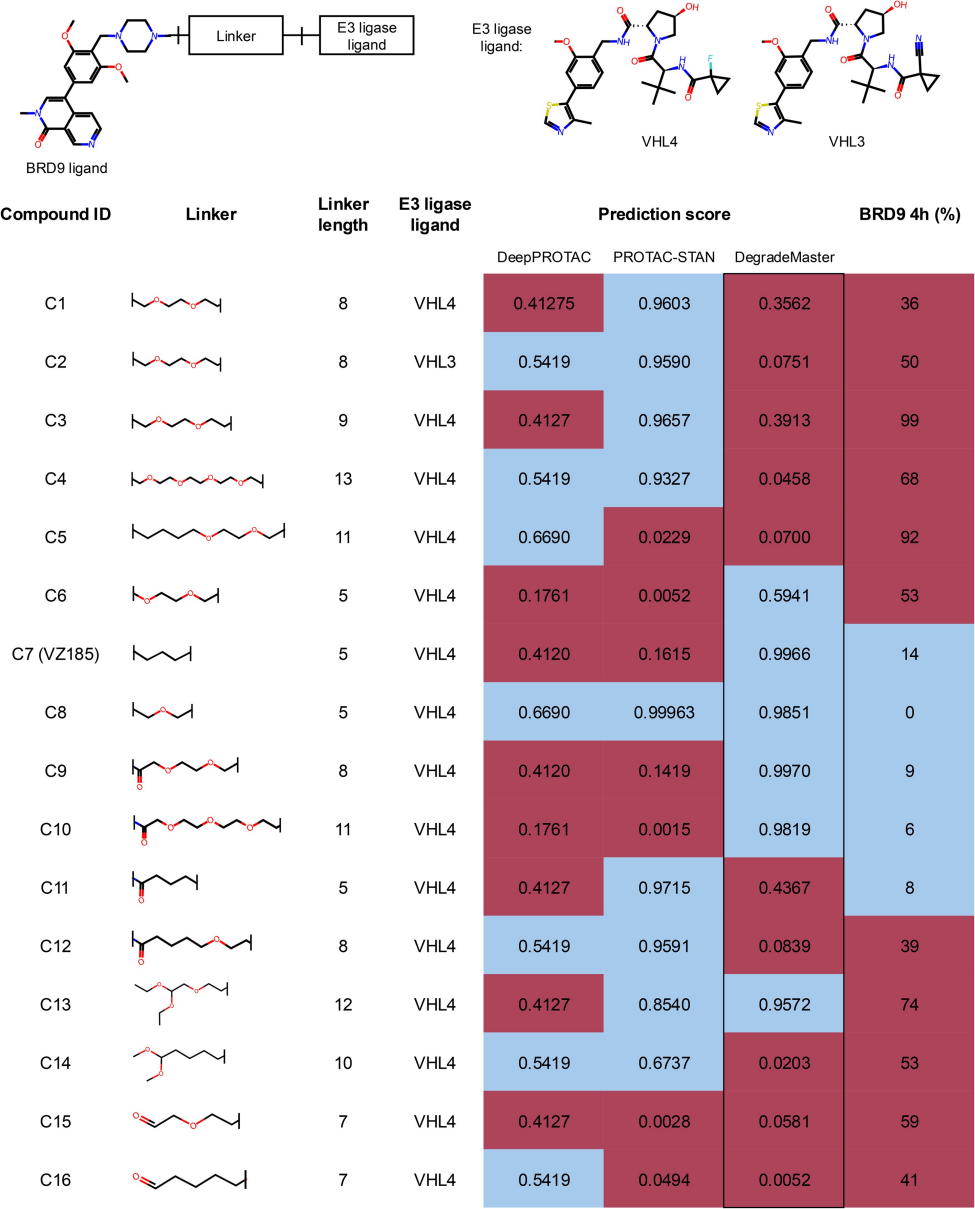
Case study 1: degradability prediction of the VZ185 (Zoppi *et al.* 2018) PROTAC candidates. Compounds 13–16 only consist of a linker and an E3 binder, with no target binder included. The rightmost column represents the degradation activity, expressed as the percentage of total protein remaining after treatment with 1 µM compound. Prediction scores range from 0 to 1, with higher scores indicating a greater likelihood of protein degradation. Threshold of high degradation (blue) and low degradation (red): 0.5 for prediction scores and 15% for protein remaining.

**Figure 6.**
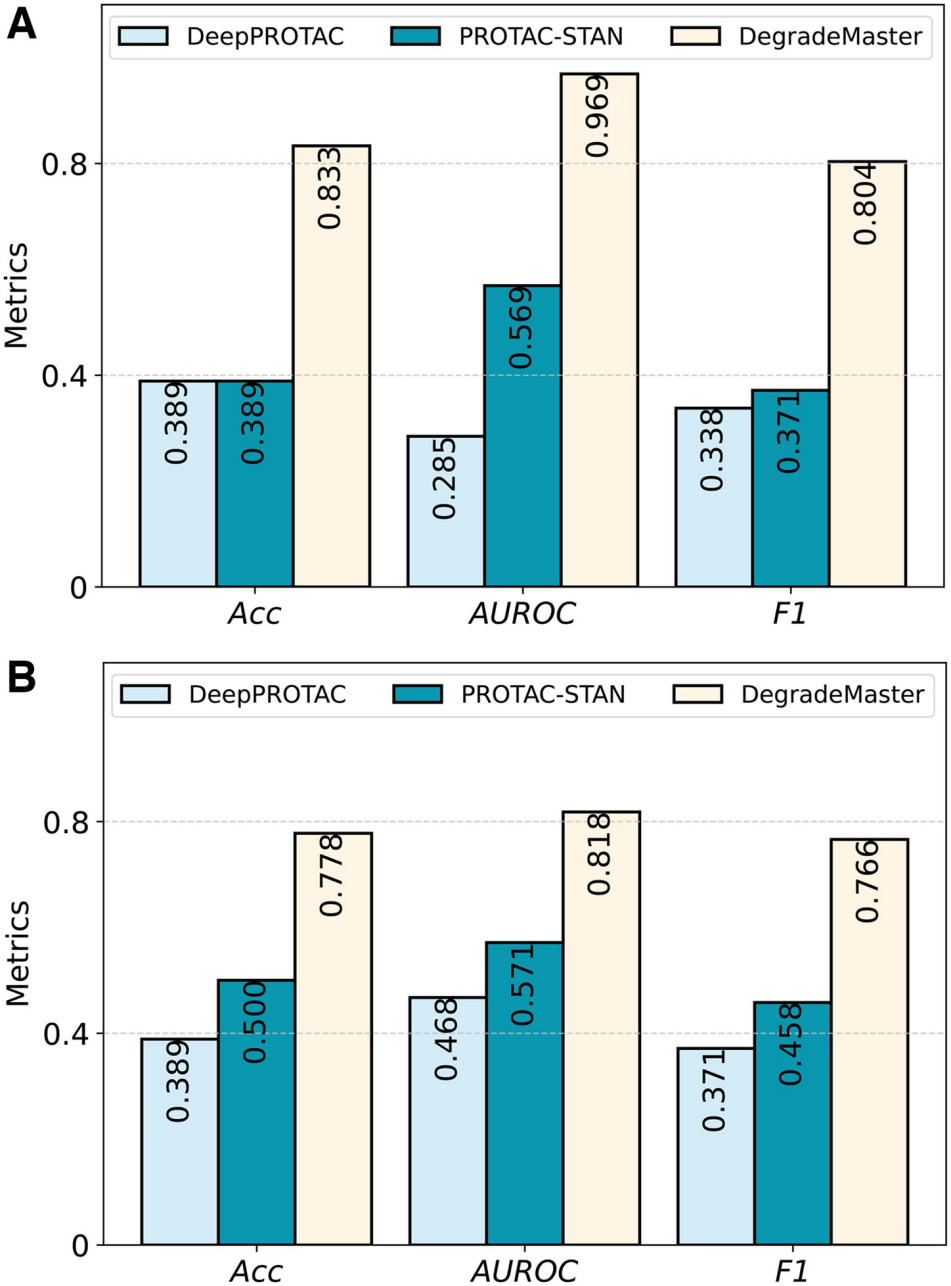
Performance comparison of DegradeMaster associated with two DL models on Case study 1 (A) and Case study 2 (B).

In Case study 1, we constructed an experimental dataset comprising 16 PROTAC candidates developed during the design of PROTAC VZ185 (Zoppi *et al.* 2018), which recruits VHL ligase to degrade BRD9, a chromatin binding factor that plays a crucial role in regulating gene expression. The dataset includes various linear alkyl chains and polyethylene glycol chains of different lengths as linkers, paired with two variants of the VHL E3 ligase binding ligand (VHL3 and VHL4), resulting in 16 PROTAC candidates. As shown in Fig. 5, the rightmost column indicates the actual performance of these PROTAC candidates. Of the 16, five exhibit high BRD9 degradation, while the remaining 11 have relatively low efficacy. Figure 6A illustrates that DegradeMaster achieves 83.33% accuracy and an AUROC of 0.9692 in predicting the BRD9 degradation by these PROTAC candidates, significantly outperforming two baseline models. Notably, as shown in Fig. 5, DegradeMaster demonstrates accurate predictions for both positive and negative samples, with only two false-positive predictions. These results highlight the potential of DegradeMaster to accelerate PROTAC development by nominating and screening promising candidates, thereby streamlining iterative the drug discovery process.

### 4.2 Case study 2

Mutations in the Kirsten rat sarcoma viral oncogene homolog (KRAS) GTPase protein are among the most prevalent in cancer. Developing small-molecule inhibitors or PROTACs capable of targeting oncogenic KRAS alleles remains challenging, with most progress limited to KRAS with a glycine to cysteine mutation at position 12 (G12C), for which specific small molecules with covalent warheads have been developed. A recent study (Popow *et al.* 2024) successfully developed a “universal” pan KRAS degrader, ACBI3, a heterobifunctional small molecule that utilizes a noncovalent KRAS ligand to bind and potently degrade 13 out of the 17 most prevalent oncogenic KRAS alleles (Popow *et al.* 2024).

In Case study 2, we evaluated the degradability of KRAS by ACBI3 (Popow *et al.* 2024) on 17 mutants, presented in Fig. 6 and Supplementary Fig. S2. DegradeMaster demonstrates superior performance across all three metrics compared to two baseline models. Notably, since the only variable among the 17 samples is the residue point mutation present in the POI sequence, these results highlight DegradeMaster’s capability to effectively capture both structural and sequence information of the POI. This further validates the potential of DegradeMaster to drive the development of novel PROTAC candidates against even highly mutagenized POI targets.

## 5 Model efficiency

**Figure 7.**
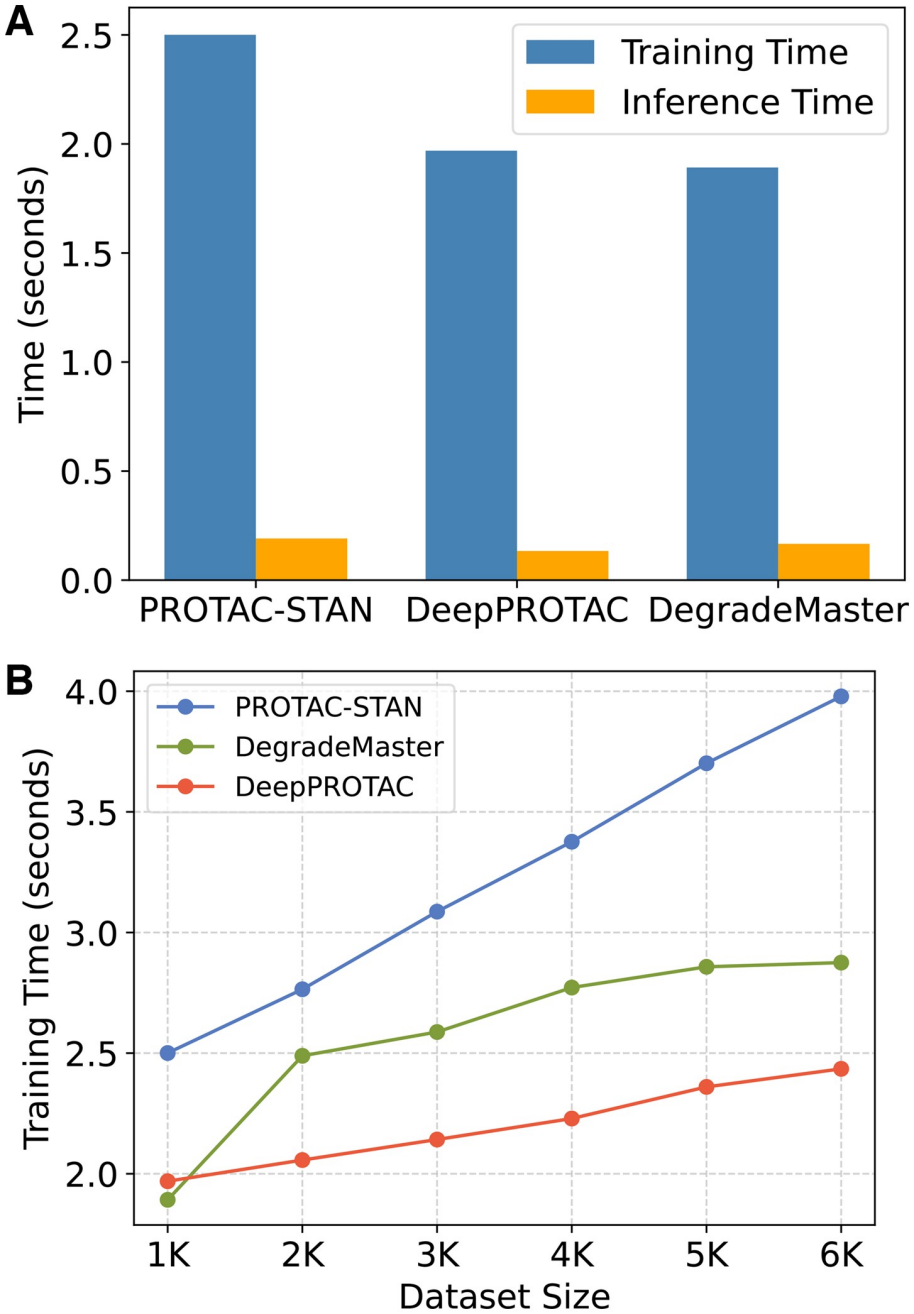
(A) Training and inference time per epoch for DeepPROTAC, PROTAC-STAN, and DegradeMaster with 1K of training samples. (B) The curve of training time when varying the dataset size from 1K to 6K.

This section compares the computational efficiency of DegradeMaster with two existing models. For a fair comparison, we set the hidden dimension as 128 and batch size as 50 for all three models. Figure 7 shows that DegradeMaster has similar training time to DeepPROTAC for 1K samples but slightly higher inference time. As dataset size grows, DeepPROTAC is 1.2× faster and consumes less memory usage (Table 4), due to its simpler 2D graph modeling and GCN encoding design. However, this simplicity comes at the cost of predictive performance, with DeepPROTAC showing a 23% lower AUROC compared to DegradeMaster. Compared to PROTAC-STAN, DegradeMaster is 1.2× faster in training, uses 27% less memory for training, and 86% less for inference. Additionally, DegradeMaster scales more efficiently with dataset size due to its lightweight mutual attention design, avoiding the computational overhead of PROTAC-STAN’s ternary attention networks.

**Table 4.** Comparison of training and inference memory usage across three models: DeepPROTAC, PROTAC-STAN, and DegradeMaster.

Mode	PROTAC-STAN	DeepPROTAC	DegradeMaster
Training	31 277 MB	4081 MB	22 757 MB
Inference	11 513 MB	1509 MB	1613 MB

## 6 Conclusion

This study introduces DegradeMaster, a semisupervised E(3)-equivariant graph neural network designed to predict PROTAC-targeted degradation with enhanced performance. By integrating 3D geometric constraints through an E(3)-equivariant encoder, employing a memory-based pseudolabeling for label enrichment, and utilizing a mutual attention pooling module for comprehensive graph embedding, DegradeMaster addresses the limitations of spatial information loss and label scarcity in existing approaches. Experiments demonstrate its superior performance over state-of-the-art methods and high accuracy in case studies involving challenging degradability predictions. Although DegradeMaster has demonstrated superior performance, classifying PROTACs into binary “high” or “low” activity remains somewhat arbitrary. This approach may overestimate the number of seemingly “active” candidates, potentially limiting DegradeMaster ’s practical applicability. In future research, we will focus on developing a regression-based model to predict more granular ranges of $DC_{50}$ and *D*_max_ values, addressing this limitation.

## Supplementary Material

btaf191_Supplementary_Data

## Data Availability

The source code and datasets are available at https://github.com/ABILiLab/DegradeMaster and https://zenodo.org/records/14715718.
