# Supplementary material for: Accurate PROTAC-targeted degradation prediction with DegradeMaster
Source: Bioinformatics. 2025 Jul 15;41(Suppl 1):i342–51. doi: 10.1093/bioinformatics/btaf191 (PMC12261415; doi:10.1093/bioinformatics/btaf191)
Supplement: btaf191_Supplementary_Data [file btaf191_supplementary_data.pdf]

Accurate PROTAC targeted degradation prediction with DegradeMaster: Supplementary

JIE LIU, MICHAEL ROY, LUKE ISBEL AND FUYI LI

1. FEATURE SELECTION

In addition to spatial and structural information, the intrinsic physicochemical properties of molecules can provide valuable insights into degradation. This section introduces our feature selection pipeline to identify the most informative chemical properties. As shown in Table S1, we extract 11 chemical properties of PROTAC molecules from PROTAC-DB 3.0. Directly inputting all raw attributes into the deep learning model risks introducing noise from irrelevant features, potentially reducing predictive performance. To address this, we implement a feature selection module with two steps, using only training set data. The first step employs a filter selection approach based on statistical metrics, including variance, Pearson correlation, chi-square score, p-value, and mutual information score, defined as follows:

- **Variance**: A metric measuring the spread of feature values from the mean. Features with very low variance are often irrelevant for distinguishing between target classes. Here we set the filtering threshold as 0.01.
- **Pearson Correlation Coefficient**: A metric measuring the strength and direction of a linear relationship between two variables, ranging from -1 (perfect negative relationship) to 1 (perfect positive relationship), with 0 indicating no linear relationship. The threshold is set as ± 0.9.
- **Chi-Square Score**: A statistical test used to evaluate the independence between features and the label. Higher scores indicate stronger associations. We set the filtering threshold as 10.
- **P-Value**: A metric that assesses the significance of a feature in hypothesis testing. Lower p-values suggest that the feature is significantly related to the label. The threshold is set as 0.05.
- **Mutual Information**: A metric that measures the amount of information shared between the feature and the label. Higher mutual information indicates a stronger relationship.

As shown in Table S1, the variance of Gasteiger charges is significantly lower than that of other properties, leading to its exclusion. The correlation matrix for all 11 molecular properties is presented in Table S2, revealing that exact mass and heavy atom count are highly correlated with molecular weight. Consequently, these two properties are filtered as redundant. Based on the chi-square score and p-value columns, we also exclude hydrogen bond acceptor count and chiral centres. However, most properties exhibit low mutual information scores, suggesting that mutual information may not be a reliable metric for feature selection in this context.

Following the filter method, we apply an embedded method for further refinement. Specifically, we use Gradient Boosting Decision Trees (GBDT) [1] as the base model, training it with the filtered features and labels to compute feature importance as the normalized total reduction in the splitting criterion. Table S1 reports the results, where hydrogen bond donor count and rotatable bond count show the lowest importance. Nevertheless, given their high chi-square scores, these descriptors are retained, underscoring the importance of integrating multiple statistical metrics in feature selection.

In conclusion, the two-step feature selection process resulted in the preservation of six chemical properties: molecular weight, XLogP3, ring count, hydrogen bond donor count, rotatable bond count, and topological polar surface area as the final selected features for PROTAC molecules. For the POI and E3 ligase proteins, we adopt the auto-cross covariance (ACC) method, as described

Chemical properties	Variance	Chi-square score	P-value	Mutual-info score	GBDT importance	Selection
Molecule weight	28345.75	10.04	0.0015	0.0720	0.2260	✓
Exact mass	28287.18	10.08	0.0014	0.0950	–	
XLogP3	5.96	13.77	0.0002	0.0778	0.2029	✓
Heavy Atom Count	130.56	0.1821	0.6695	0.0186	–	
Ring Count	1.83	20.63	≈0	0.0537	0.1928	✓
Hydrogen Bond Acceptor Count	8.85	0.0650	0.7987	0.0226	–	
Hydrogen Bond Donor Count	2.87	26.95	≈0	0.0055	0.0556	✓
Rotatable Bond Count	40.71	70.78	≈0	0.0071	0.0551	✓
Topological Polar Surface Area	2004.48	340.9	≈0	0.1764	0.2673	✓
Gasteiger charges	0.01	0.0034	0.9533	0.1844	–	
Chiral centers	3.47	1.241	0.2651	0.0133	–	

Table S1. Chemical properties of PROTAC molecules collected from PROTAC-DB 3.0 and their corresponding statistical metrics.

	Molecular Weight	Exact Mass	XLogP3	Heavy Atom Count	Ring Count	Hydrogen Bond Acceptor Count	Hydrogen Bond Donor Count	Rotatable Bond Count	Topological Polar Surface Area	Charges	Chiral Centers
Molecular Weight	nan	**0.9999**	0.4694	**0.9871**	0.3956	0.6233	0.4244	0.7538	0.6047	0.2627	0.5167
Exact Mass	nan	nan	0.4691	**0.9873**	0.3958	0.6236	0.4246	0.7539	0.6051	0.2627	0.5167
XLogP3	nan	nan	nan	0.4620	0.2595	-0.1457	-0.0051	0.2262	-0.2374	0.2851	0.3898
Heavy Atom Count	nan	nan	nan	nan	0.4677	0.6283	0.4029	0.7376	0.6061	0.2557	0.5086
Ring Count	nan	nan	nan	nan	nan	0.2880	-0.2365	-0.0587	0.0424	0.0365	0.0882
Hydrogen Bond Acceptor Count	nan	nan	nan	nan	nan	nan	0.2694	0.6372	0.7310	0.1547	0.1128
Hydrogen Bond Donor Count	nan	nan	nan	nan	nan	nan	nan	0.4980	0.7296	-0.0199	0.3532
Rotatable Bond Count	nan	nan	nan	nan	nan	nan	nan	nan	0.6444	0.1370	0.3203
Topological Polar Surface Area	nan	nan	nan	nan	nan	nan	nan	nan	nan	-0.0195	0.2277
Charges	nan	nan	nan	nan	nan	nan	nan	nan	nan	nan	0.2477
Chiral Centers	nan	nan	nan	nan	nan	nan	nan	nan	nan	nan	nan

Table S2. Correlation matrix for molecular descriptors, computed using pearson correlation method. The correlations above the threshold 0.9 are in bold.

in [2], to represent protein sequence features. ACC quantifies the relationship between amino acid properties within a protein sequence across varying lags.

2. PERFORMANCE EVALUATION METRICS

To assess the performance of DegradeMaster and its competitors, we utilize a combination of ROC-AUC, precision, recall, and Macro-F1 as evaluation metrics. ROC-AUC, a standard metric in anomaly detection, involves plotting the true positive rate against the false positive rate, with AUC (Area Under the Curve) representing the area under this ROC curve, ranging between 0 and 1. Higher AUC values indicate better performance. Additionally, we use precision, recall, and macro-F1 as our evaluation metrics. For each class, the precision, recall, and F_1 score can be calculated as follows:

$$Precision = \frac{TP}{TP + FP},$$

$$Recall = \frac{TP}{TP + FN},$$

$$F_1 = 2 \times \frac{Precision \times Recall}{Precision + Recall},$$

where TP denotes the number of positive samples that are correctly predicted; FP is the number of negative samples that are predicted as the positive ones; and FN is the number of positive samples that are predicted as the negative ones. Macro-F1, as the harmonic mean of precision and recall, is computed individually for each class and then averaged, to provide a balanced measure of the model's performance. Therefore, by averaging the F_1 scores of both classes, macro-F1 can be obtained as follows:

$$Macro\text{-}F1 = \frac{F_1(L) + F_1(H)}{2},$$

where L and H denote the low-activity and the high-activity class, respectively.

3. TRAINING SETTING

In this section, we will introduce the data leakage control strategy, hyper-parameter tuning process, and final training process in detail.

Firstly, we will clarify the strategies used to prevent data leakage when spliting datasets. As stated in the manuscript, we split both the PROTAC-1K and PROTAC-8K datasets into training and test sets using a random 80/20 division. It is true that some identical components may appear in both the training and test sets. To avoid data leakage, we removed overlapping data samples from the test set. Specifically, if a sample in the test set has the same target protein and PROTAC SMILES as a sample in the training set, we exclude it from the test set. As demonstrated in [3, 4], two structurally similar PROTACs differing by only a few atoms in the linker can exhibit significantly different degradation activities. Additionally, as shown in [5], the same PROTAC can display varying degradation effects depending on the target protein. Therefore, compared to PROTAC-STAN, which excludes all test set samples if a PROTAC appears in the training set, our approach is more reasonable. It removes exact duplicates while retaining potentially structurally similar yet distinct molecules, enabling the model to generalize beyond memorization while still assessing its predictive capability on related but novel compounds. Besides, our strategy also helps validate the model's ability to predict the degradability of the same PROTAC on different target proteins.

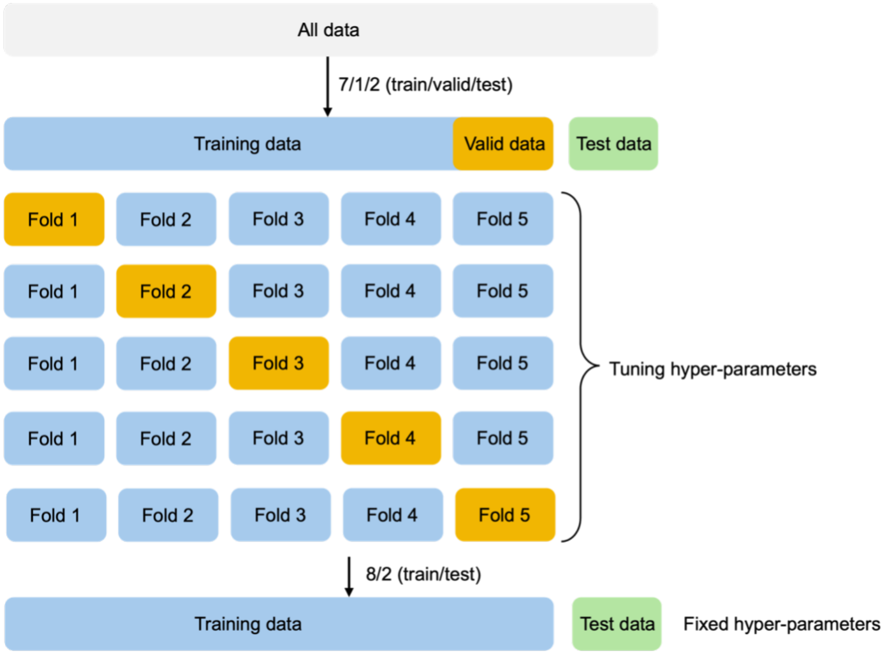

Fig. S1. Illustration of dataset split and training process

Secondly, as decipted in Figure S1, we first randomly split the whole dataset as 7/1/2 for train/validation/test sets. During hyper-parameter tuning, the test set was held out, and we

Random seed	DegradeMaster-*Semi*			DegradeMaster-*Super*		
	Acc (%)	AUROC	F1 score	Acc (%)	AUROC	F1 score
Seed=63	84.58	0.9050	0.8394	81.41	0.8350	0.7975
Seed=64	87.09	0.8868	0.8584	81.85	0.8467	0.7947
Seed=65	86.09	0.9084	0.8546	83.58	0.8761	0.8138
Seed=66	85.48	0.8961	0.8390	80.86	0.8356	0.8059
Seed=111	83.66	0.8825	0.8224	81.41	0.8541	0.8139
Average	85.38	0.8958	0.8428	81.82	0.8495	0.8052
Variance	1.4073	0.0001	0.0002	0.8713	0.0002	0.0001

Table S3. Model performance with different random seeds, the rightmost column shows the average performance and standard variances of the five experiments.

applied 5-fold cross validation to the remaining 80% of the whole data. Specifically, the data is split into 5 folds, and each fold is used once as the validation set while the remaining 4 folds are used for training. The model is trained and validated on each fold, and the average results are reported across all folds. The hyper-parameters were chosen based on the model performance on validation set. This ensures our model is trained and tuned in robustness across different data distribution. The tuned hyper-parameters are reported in Table S4. After the hyper-parameter tuning, we merge the training and validation sets and create a new training set which consists of 80% of the overall data. The test set remained unchanged. Then we fix the hyper-parameters and train the model on the training set, and evaluate it on the test set. It can be seen that the test set remains independent from the training and validation sets for the whole time.

To further demonstrate the robustness of DegradeMaster, and that the results are not contingent on a specific dataset split, we chose 5 different random seeds, randomly shuffled the dataset, and repeated the entire process shown in Figure S1 for 5 times. The corresponding results are shown in Table S3.

Among the five random seeds, the results for "seed=111" were those reported in the original paper. As shown in Table S3, DegradeMaster consistently achieved superior performance across all five dataset splits, as well as in the averaged results. Additionally, the model exhibited generally low performance variance across different dataset splits. These findings DegradeMaster's superior performance stems from its robust representation and predictive capabilities, rather than being influenced by a specific data split.

4. MODEL COMPARISON WITH PROTAC-STAN

In this section, we will discuss about the key differences between DegradeMaster and the state-of-the-art baseline PROTAC-STAN. There are three major differences between DegradeMaster and the most competitive baseline, PROTAC-STAN. First, regarding the encoder framework, PROTAC-STAN processes protein sequences using a protein language model (PLM) without explicitly incorporating protein structure data. While this simplifies preprocessing, it fails to capture essential spatial dependencies due to the lack of explicit 3D structural modeling, leading to a loss of critical spatial relationships. Additionally, for PROTAC encoding, PROTAC-STAN employs a 2-layer GCN, which models molecules as 2D graphs, further disregarding their spatial properties. In contrast, DegradeMaster utilizes E(3)-equivariant encoders to explicitly encode geometric constraints in both molecule and protein representations, significantly improving predictive performance, as demonstrated in Section 3.2. Second, a major distinction lies in the attention mechanism design. Both models employ attention modules to integrate POI, PROTAC, and E3 embeddings; however, PROTAC-STAN utilizes a ternary attention network, which computes a dense ternary attention map $\mathbf{A} \in \mathbb{R}^{\alpha\times\beta\times\gamma}$ (where α, β, γ represent the number of POI, PROTAC and E3 embeddings, respectively), This approach imposes a significant computational burden, limiting PROTAC-STAN's scalability to larger batch sizes, as validated in Section 5. In contrast, DegradeMaster employs a mutual attention module, which computes attention weights separately between node-level embeddings and graph-level features within POI, PROTAC, and E3 graphs. This results in three significantly smaller attention maps: $\mathbf{A}_1 \times \mathbb{R}^{\alpha\times 1}$, $\mathbf{A}_2 \times \mathbb{R}^{\beta\times 1}$, $\mathbf{A}_3 \times \mathbb{R}^{\gamma\times 1}$, drastically reducing computational and memory overhead. Finally, the third key

Category	Hyper-parameter	Value	Description
Model	Hidden size	128	Dimension of hidden layer of E(3) equivariant encoder
	Number of layers	2	Number of hidden layers of E(3) equivariant encoder
	K	1000	Number of pseudo labels for each class
	PROTAC feature	172	Dimension of PROTAC attributive features
	PROTAC embedding	118	Dimension of PROTAC embeddings
	E3 ligase feature	30	Dimension of E3 ligase attributive features
	POI feature	30	Dimension of POI attributive features
Training	Pre-train learning rate	0.0001	Learning rate in pre-train
	Re-train learning rate	0.001	Learning rate in re-train
	Pre-train epoch	30	Number of iterative times in pretraining DegradeMaster
	Re-train epoch	2000	Number of iterative times in retraining DegradeMaster
	Batch size	40	Number of samples per step
	Test ratio	0.2	Test set ratio of the entire dataset
	Start epoch	0	The epoch that starts for calculating disagreement score
	End epoch	20	The epoch that ends for disagreement score

Table S4. Summary of hyperparameters and configuration.

difference is in the pseudo-labeling strategy. As discussed in Section 3.1, PROTAC-STAN was originally designed as a fully supervised model, limiting its ability to leverage unlabelled data. In contrast, DegradeMaster incorporates pseudo-labeling, enabling it to utilize abundant unlabelled data during training, further enhancing its predictive performance.

5. QUALITY CONTROL IN DATASET CONSTRUCTION

In this section, we provide additional details on the construction of the PROTAC-1K and PROTAC-8K datasets. Among all protein structures used, 91% are crystal structures obtained from the PDB database, while the remaining 9% are derived from AlphaFold 2 predictions. Specifically, crystal structures were unavailable in the PDB for 38 out of 442 target proteins and 3 out of 22 E3 ligases. For these proteins, we utilized AlphaFold 2 (AF2) for structure prediction. To ensure the quality of the predicted structures, we used the pLDDT score provided by AF2 as a confidence measure. pLDDT assesses the reliability of predicted atomic positions within a protein structure on a scale of 0 to 100, with values above 90 indicating high reliability. Therefore, for AF2-predicted structures, we retained only residues with very high confidence (pLDDT > 90).

Regarding the quality control of AutoDock Smina, we performed Smina docking 10 times for each ligand-protein pair and selected the result with the lowest affinity score. On average, the best docking scores were below -7 kcal/mol, indicating strong ligand-protein binding. Additionally, we ensured that all PROTACs included in the two case studies—including the 16 PROTAC candidates for VZ185 and ACBI3—were not present in the training or validation sets.

REFERENCES

1. J. H. Friedman, “Greedy function approximation: a gradient boosting machine,” Annals statistics pp. 1189–1232 (2001).
2. F. Li, Q. Hu, X. Zhang, *et al.*, “Deepprotacs is a deep learning-based targeted degradation predictor for protacs,” Nat. communications **13**, 7133 (2022).
3. V. Zoppi, S. J. Hughes, C. Maniaci, *et al.*, “Iterative design and optimization of initially inactive proteolysis targeting chimeras (protacs) identify vz185 as a potent, fast, and selective von hippel–lindau (vhl) based dual degrader probe of brd9 and brd7,” J. medicinal chemistry **62**, 699–726 (2018).
4. R. I. Troup, C. Fallan, and M. G. Baud, “Current strategies for the design of protac linkers: a critical review,” Explor. Target. Anti-tumor Ther. **1**, 273 (2020).

5. K. Li and C. M. Crews, “Protacs: past, present and future,” Chem. Soc. Rev. **51**, 5214–5236 (2022).
6. J. Popow, W. Farnaby, A. Gollner, *et al.*, “Targeting cancer with small-molecule pan-kras degraders,” Science **385**, 1338–1347 (2024).

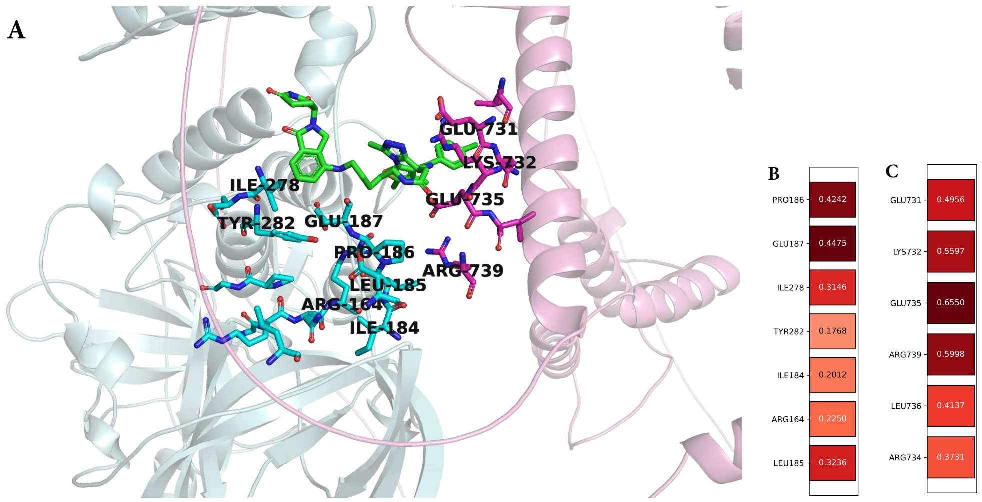

Fig. S2. (**A**) Visualization of the ternary complex comprising the PROTAC molecule (PROTAC-DB ID: 194), POI, and E3 ligase. The E3 ligase is shown in cyan, and the POI is depicted in light pink. The 5 Å protein pockets of both the E3 ligase and POI are further visualized, with residue names labeled. The PROTAC molecule is represented in green. (**B**) Attention weight distribution for residues within the E3 ligase pocket. (**C**) Attention weight distribution for residues within the POI pocket. The attention weight of each residue is computed as the average of the attention weights assigned to all atoms within the residue.

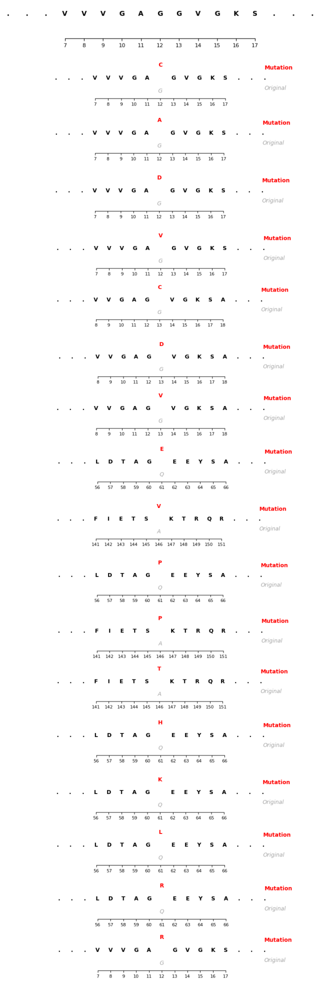

Mutant ID	Mutant Sequence	Mutation	PROTAC	Prediction			Degradability
				DeepPROTAC	PROTAC-STAN	DegradeMaster	
1		None	ACBI3	0.3172	0.9876	0.9379	$DC_{50} \leq 100nM$
2		G12C	ACBI3	0.5419	0.9743	0.1936	$DC_{50} \leq 100nM$
3		G12A	ACBI3	0.3168	0.9875	0.9382	$DC_{50} \leq 100nM$
4		G12D	ACBI3	0.5419	0.9752	0.1945	$DC_{50} \leq 100nM$
5		G12V	ACBI3	0.6690	0.9998	0.9801	$DC_{50} \leq 100nM$
6		G13C	ACBI3	0.6690	0.9998	0.9801	$DC_{50} \leq 100nM$
7		G13D	ACBI3	0.6361	0.0047	0.9982	$DC_{50} \leq 100nM$
8		G13V	ACBI3	0.1761	0.0067	0.8824	$DC_{50} \leq 100nM$
9		Q61E	ACBI3	0.2077	0.1675	0.9946	$DC_{50} \leq 100nM$
10		A146V	ACBI3	0.1761	0.0069	0.8828	$DC_{50} \leq 100nM$
11		Q61P	ACBI3	0.3180	0.9879	0.9374	$DC_{50} \leq 100nM$
12		A146P	ACBI3	0.3172	0.9876	0.9384	$DC_{50} \leq 100nM$
13		A146T	ACBI3	0.5419	0.9728	0.0352	$DC_{50} \leq 100nM$
14		Q61H	ACBI3	0.5419	0.1222	0.1922	$DC_{50} > 100nM$
15		Q61K	ACBI3	0.3163	0.9766	0.9379	$DC_{50} > 100nM$
16		Q61L	ACBI3	0.5196	0.9875	0.0323	$DC_{50} > 100nM$
17		Q61R	ACBI3	0.5419	0.1190	0.1958	$DC_{50} > 100nM$
18		G12R	ACBI3	0.5420	0.9743	0.1936	$DC_{50} > 100nM$

Fig. S3. Case study 2: degradation prediction of the ACBI3 [6] on KRAS mutants. Protein 1 has no mutations whereas the others have a mutated residue at the corresponding position. The rightmost column represents the degradation activity, expressed as the DC_{50} values. Prediction scores range from 0 to 1, with higher scores indicating a greater likelihood of protein degradation. Threshold of high degradation (blue) and low degradation (red): 0.5 for prediction scores and 100 nM for DC_{50}.

A

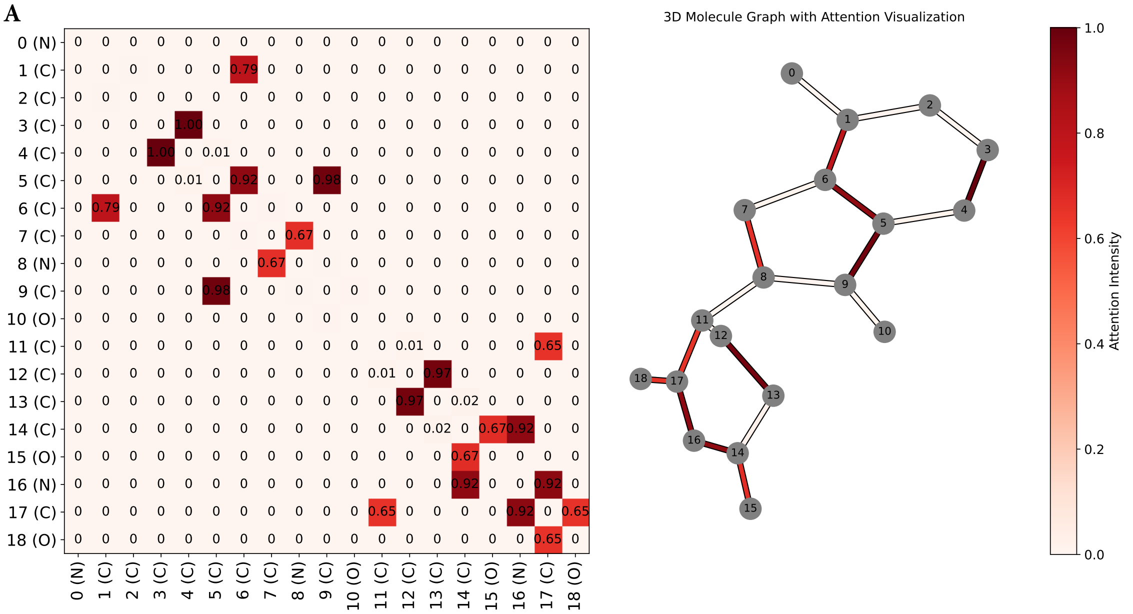

Fig. S4. Visualization of attention weights for the E3 ligand of PROTAC molecule (PROTAC-DB ID: 194), including the complete attention matrix.

B

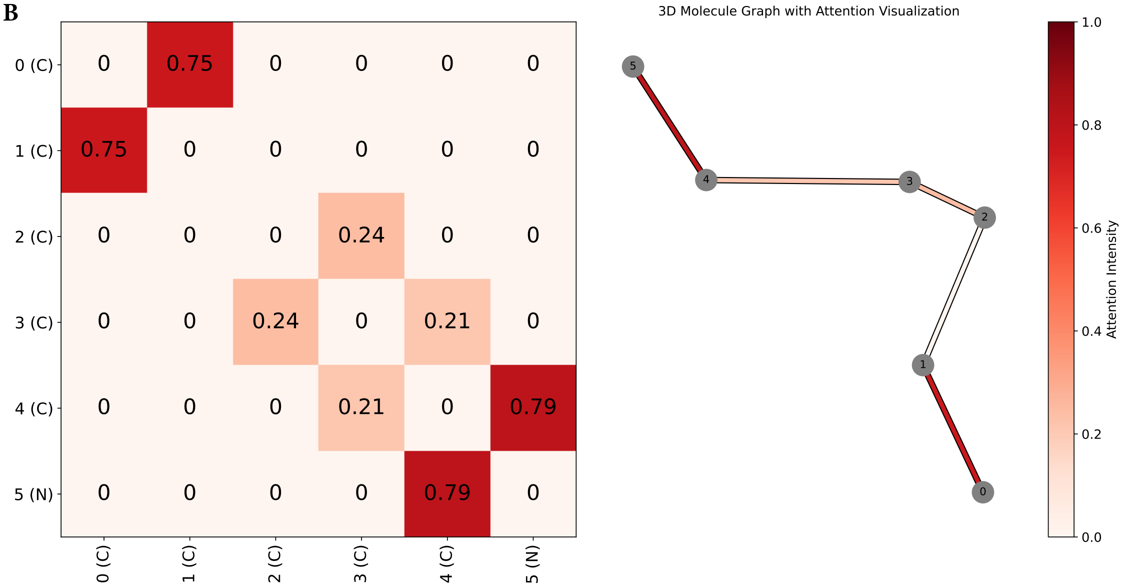

Fig. S5. Visualization of attention weights for the linker of PROTAC molecule (PROTAC-DB ID: 194), including the complete attention matrix.

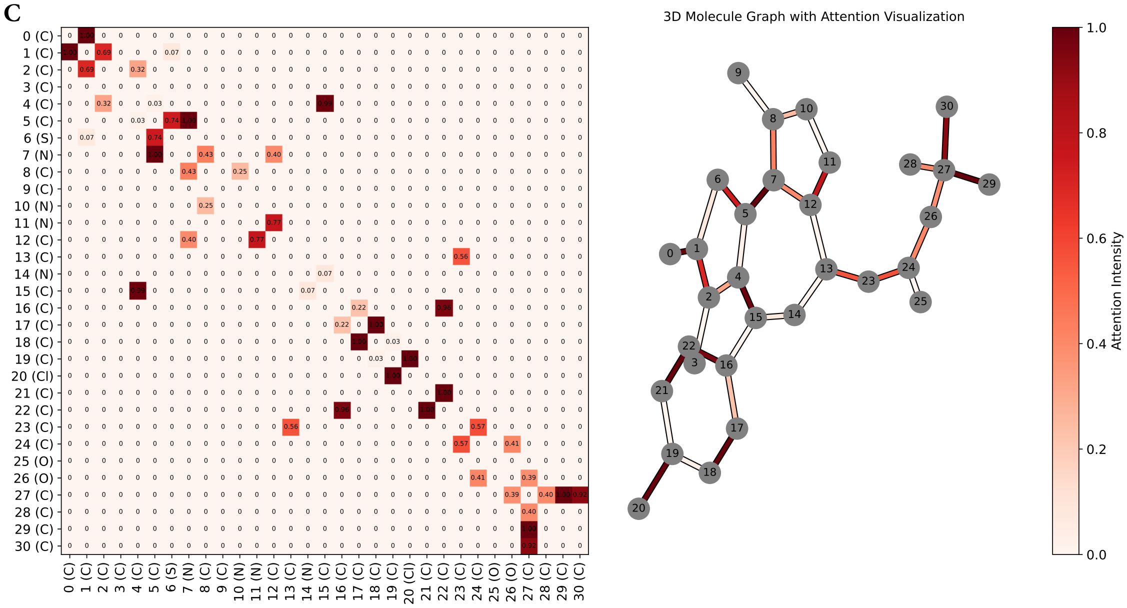

Fig. S6. Visualization of attention weights for the warhead of PROTAC molecule (PROTAC-DB ID: 194), including the complete attention matrix.

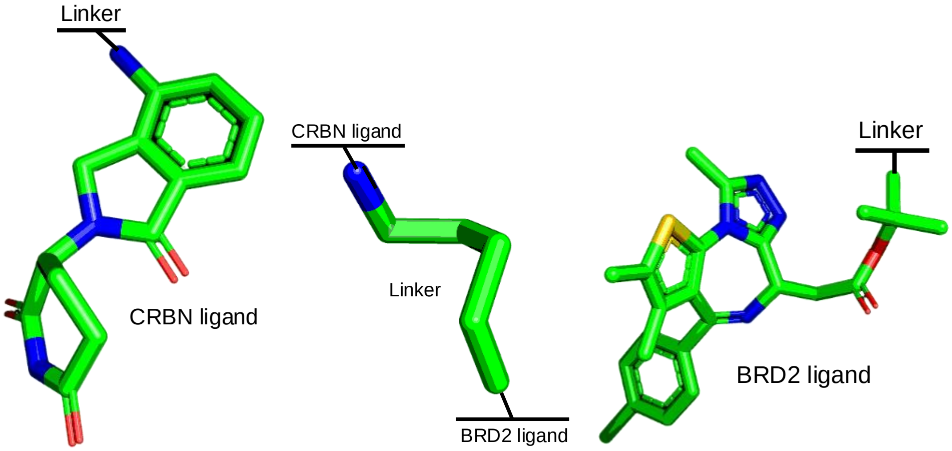

Fig. S7. The chemical structures of the E3 ligand, linker, and warhead of PROTAC-194.
